# Prosthesis Tailoring for Patients Undergoing Transcatheter Aortic Valve Implantation

**DOI:** 10.3390/jcm12010338

**Published:** 2023-01-01

**Authors:** Pier Pasquale Leone, Andrea Scotti, Edwin C. Ho, Manaf Assafin, James Doolittle, Mei Chau, Leandro Slipczuk, Matthew Levitus, Damiano Regazzoli, Antonio Mangieri, Azeem Latib

**Affiliations:** 1 Montefiore-Einstein Center for Heart and Vascular Care, Montefiore Medical Center, Albert Einstein College of Medicine, Bronx, NY 10461, USA; 2 Department of Biomedical Sciences, Humanitas University, 20072 Pieve Emanuele, Italy; 3 Cardio Center, IRCCS Humanitas Research Hospital, 20089 Rozzano, Italy; 4 Cardiovascular Research Foundation, New York, NY 10019, USA

**Keywords:** TAVI, TAVR, aortic stenosis, transcatheter heart valve, prosthesis

## Abstract

Transcatheter aortic valve implantation (TAVI) has risen over the past 20 years as a safe and effective alternative to surgical aortic valve replacement for treatment of severe aortic stenosis, and is now a well-established and recommended treatment option in suitable patients irrespective of predicted risk of mortality after surgery. Studies of numerous devices, either newly developed or reiterations of previous prostheses, have been accruing. We hereby review TAVI devices, with a focus on commercially available options, and aim to present a guide for prosthesis tailoring according to patient-related anatomical and clinical factors that may favor particular designs.

## 1. Introduction

Aortic stenosis (AS) represents the most common valvular heart disease in developed countries. Almost one out of ten individuals between 80 and 89 years old are affected, and the number of elderly patients with non-rheumatic, calcific AS is projected to more than double by 2050 [1,2,3]. The disease course, once symptoms arise, is inevitable if left untreated [4]. Surgical aortic valve replacement (SAVR) has long been the only available treatment, until transcatheter aortic valve implantation (TAVI) was introduced in 2002 [5]. In the last twenty years, the patient population undergoing treatment [6,7,8,9,10,11,12,13,14,15] and the procedure itself [16] have changed tremendously, such that TAVI is now indicated in patients with symptomatic severe AS deemed suitable for the procedure, irrespective of their predicted risk of mortality after SAVR, when at least 65 [17] or 75 years of age [18]. Numerous tri-leaflet prostheses have been developed, and are characterized by specific design elements, including different frames, mechanisms of expansion, leaflet material and position that may affect their suitability in certain situations [19].

We aim to review TAVI prostheses, either currently available or under clinical investigation, and the key demographic and anatomical factors to consider when tailoring prosthesis choice to different scenarios encountered in the clinical setting, while acknowledging each operator’s responsibility for adherence to good clinical practice and device indications.

## 2. Prostheses

**Figure 1:**
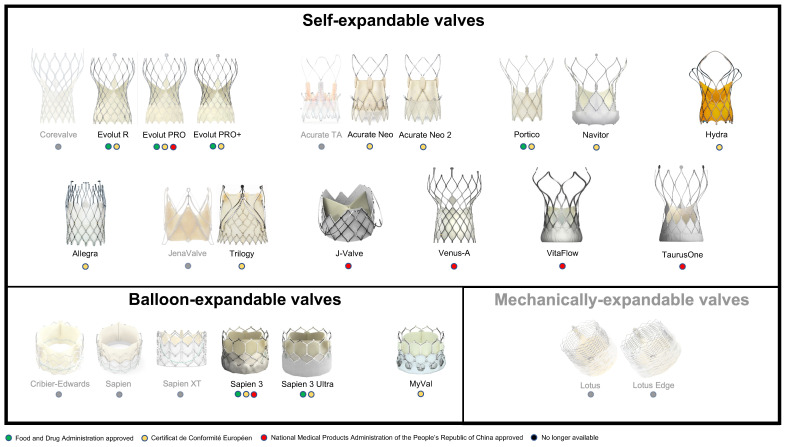
**Families of bioprostheses for TAVI.** The more transparent transcatheter heart valves represent no longer available bioprostheses. TAVI = transcatheter aortic valve implantation.

The main design classification applied to TAVI prostheses is defined according to the mechanism of valve expansion, and includes self-expanding valves (SEV), balloon-expandable valves (BEV) or mechanically expandable valves (MEV). The greatest clinical experience with TAVI worldwide has been with SEV and BEV, with SEV representing the most diverse group. On the other hand, MEV have been less represented in the prosthetic landscape, and production of the only commercial MEV has now ceased. Overall, not only have revisions of the earliest prostheses been developed over the years, but also different new technologies have recently entered the market (Figure 1). We hereby collected relevant information on each individual family of prostheses, focusing on the latest iteration when available.

### 2.1. Self-Expandable Valves

#### 2.1.1. Evolut PRO+ and CoreValve Family

The Evolut PRO+ (Medtronic, Minneapolis, MN, USA) is the latest iteration from the CoreValve family of valves [20]. The leaflets are porcine pericardial and in a supra-annular position, which allows for optimal forward flow hemodynamics with a larger effective orifice area (EOA), lower trans-prosthetic gradients and lower risk of prosthesis-patient mismatch (PPM) compared to intra-annular valves [21]. Its largest size (34 mm) allows treatment of patients with annuli up to 30 mm in diameter, and its outer porcine pericardial wrap reduces risk of paravalvular leak (PVL). Its long (approximately 46 mm) nitinol frame yields consistent radial force, at the price of greater impingement on nearby structures, including the conduction system [22]. It is also more challenging to subsequently access the coronary arteries, since both the top of the valve frame and the prosthetic leaflet commissures often sit above the coronary ostia and the frame’s diamond shaped cells are relatively small in size (approximately 12 French) [23]. The outer diameter (OD) of the non-steerable delivery system is 14 French (for 23, 26, 29 mm valves) or 16 French (for the 34 mm valve), allowing compatibility with 18 and 20 French OD sheaths, respectively. The delivery catheter also has the EnVeo InLine^TM^ sheath that replaces the need for a separate introducer sheath, allowing a 4 French reduction in arteriotomy size and compatibility with a minimum vessel diameter of 5 mm. Replacement of the double-spine with a single-spine shaft in the recently introduced Evolut FX system improved delivery system flexibility and valve deliverability. Finally, the device is recapturable, repositionable and retrievable until partial deployment (75–80%), which roughly corresponds to when the TAVI valve starts functioning. This multi-step valve deployment process may increase contrast use, procedure and fluoroscopy time (Table 1 and Table 2).

#### 2.1.2. Acurate Neo and Acurate Neo 2

The Acurate Neo 2 (Boston Scientific, Marlborough, MA, USA) received Conformité Européen (CE) mark in 2020, while the Acurate IDE trial (NCT03735667) has an estimated primary completion date in early 2024. It shares many features with its Acurate Neo predecessor, including a self-expanding nitinol frame and porcine pericardial supra-annular leaflets. Its overall design comprising of axial stabilization arches above the leaflet level with a large open space in between them and an upper crown allows for easier coronary access and reduces risk of coronary obstruction. In addition, after higher rates of moderate or greater PVL were observed with Acurate Neo compared to other devices in two investigator-initiated randomized studies [24,25], a 60% taller integrated inner and outer pericardial skirt was introduced with the Acurate Neo 2 prosthesis. The sheath is 14 French and expandable. When compared to devices currently available from the CoreValve family, these prostheses exert less opening and radial force, potentially making predilation almost mandatory. The unique top-down deployment allows for controlled implant depth optimization, but repositionability is not possible. It comes in small, medium and large sizes [26].

#### 2.1.3. Portico and Navitor

The Portico (Abbott, Chicago, IL, USA) is the second SEV to have obtained Food and Drug Administration (FDA) approval in the United States. This occurred in 2021 for patients at high or extreme surgical risk [27]. This prosthesis uses bovine pericardial intra-annular leaflets mounted on a nitinol frame and has an overall frame height similar to the previously discussed SEV. It is recapturable, repositionable and retrievable until 80–90% deployment, and its large frame cell design (13.5–20.8 Fr according to valve size) simplifies coronary access when compared with the CoreValve family of prostheses. Similarly to Acurate Neo/Neo 2, predilation is encouraged due to its high compliance and lower radial frame force [28]. The low-profile FlexNav^TM^ delivery system (14 French equivalent for 23 and 25 mm valves, 15 French equivalent for 27 and 29 mm valves) allows for greater three-dimensional maneuverability in challenging vascular anatomy, such as tortuous iliofemoral vessels or a very horizontal aorta [29]. In order to optimize sealing at the landing zone, the annular porcine pericardial cuff was replaced by the NaviSeal^TM^ active sealing cuff [30] in its new iteration, the Navitor valve (Abbott, Chicago, IL, USA), which received CE mark in 2021. The Navitor Titan valve (35 mm), a recent addition to the originally available four valve sizes (23, 25, 27 and 29 mm), enlarged the range of treatable annuli with this valve (from 19 up to 30 mm in diameter) [31].

#### 2.1.4. Others

The Hydra (Sahajanand Medical Technologies Limited, Mumbai, India) prosthesis is designed with a high sealing skirt mounted on a high radial force nitinol stent frame that is not flared at the inflow, a central component with high hoop force and a flexible outflow structure with three tentacle-like components to optimize alignment and conformability to the aorta. The leaflets are bovine pericardial and supra-annular. Large open frame cells (≥15 French) facilitate easier coronary access. The flexible delivery system is 18 French and the valve is recapturable, repositionable and retrievable until 80% deployment [32]. It obtained CE mark in 2020.

Various domestic TAVI prostheses in China have been developed to address the relatively young population and large number with underlying bicuspid aortic valves. They share the common feature of high radial force and include the Venus-A (Venus Medtech Inc., Hangzhou, China) [33,34], VitaFlow (MicroPort, Shanghai, China) [35] and TaurusOne (Peijia Medical, Suzhou, China) [36].

Similarly to the majority of SEV, the Allegra (New Valve Technology, Hechingen, Germany) has bovine pericardial leaflets mounted within a nitinol stent. The supra-annular leaflets and associated forward-flow hemodynamics may make this prosthesis particularly appropriate for the setting of valve-in-valve (ViV) procedures for surgical aortic bioprosthetic valve failure. Its design includes a Permaflow feature, which maintains flow during implantation, with the intent to abolish the need for rapid pacing [37].

Lastly, dedicated prostheses were designed to treat patients with isolated aortic regurgitation (AR), including the Trilogy (JenaValve Technology GmbH, Munich, Germany) and J-Valve (JC Medical Inc., Burlingame, CA, USA). The Trilogy is the second iteration of the JenaValve prosthesis, and it consists of a porcine root tissue valve mounted on a nitinol frame that has three locators designed to engage on the native aortic valve leaflets. An 18 French transfemoral delivery system replaced the prior sheathless 32 French transapical delivery catheter [38,39]. Similarly, the J-Valve has three U-shaped anchor rings designed to engage the native valve leaflets to allow the SEV to deploy within the secured leaflets. Initially implanted via the transapical route, the first-in-human transfemoral implantation via an 18 French steerable delivery system was reported in 2019. The intended treatment population of these devices is both patients with AS and AR [40].

### 2.2. Balloon-Expandable Valves

#### 2.2.1. Sapien 3 Ultra and Sapien Family

The Sapien 3 and Sapien 3 Ultra (Edwards Lifesciences, Irvine, CA, USA) are the third and fourth iterations within the Sapien family, respectively, and are the only currently FDA approved BEV. Unlike most SEV, the Sapien 3 utilizes a cobalt-chromium alloy frame and intra-annular bovine pericardial leaflets. The risk of PVL is reduced by a textured polyethylene terephthalate (PET) outer cuff, which is increased in height by 40% in the Sapien 3 Ultra. The low stent frame height (15–20 mm in Sapien 3 Ultra) and large open-cell configuration of the upper frame allow for often straightforward coronary artery access. The delivery system consists of the expandable eSheath (14 and 17.4 French ID and OD, respectively, for 20, 23 and 26 mm, and 16 and 20 French ID and OD, respectively, for the 29 mm Sapien 3 Ultra), the steerable Commander delivery system, and a fine wheel adjustment knob for accurate valve alignment and positioning. Since this prosthesis is neither repositionable nor retrievable, the operator has only a single attempt at deployment [41]. RESILIA leaflet tissue anticalcification technology and independent valve rotation control enabling commissural alignment are new features included in the latest iteration Sapien X4, not commercially available yet as of October 2022.

#### 2.2.2. MyVal

Made of a nickel-cobalt alloy, the MyVal (Meril, Vapi, India) has a hybrid honey-comb shaped scaffold design similar to the Sapien family. The upper frame cells are taller and larger, while the lower cells are more tightly packed to provide higher radial force. This combined with a PET cuff reduces the risk of PVL. Its leaflets are also bovine pericardial and intra-annular. The unique feature of this prosthesis is that it is available in nine different sizes, each 1.5 mm apart ranging from 20 mm to 32 mm, which allows for treatment of patients across a wide range of annular dimensions [42]. It received CE mark in 2019.

### 2.3. Mechanically Expandable Valves

Lotus and Lotus Edge (Boston Scientific, Marlborough, MA, USA) prostheses were the only MEV available both in the US and in Europe, until the recall in late 2020 due to delivery system issues affecting the repositionability and recapturability of the prosthesis. Nonetheless, the outcomes after deployment of this valve with a braided nitinol frame, very high radial strength and adaptive seal will be discussed [43].

## 3. Factors to Consider for TAVI Prosthesis Tailoring

It is important to take into account multiple factors when planning a TAVI procedure, especially in light of the potential complications that may occur. Given the different characteristics of available transcatheter prostheses, the type of valve implanted plays a key role in addition to numerous other procedural considerations. Below we discuss how anatomic and clinical factors may influence the choice of prosthesis. In addition, ViV procedures and TAVI for isolated AR will be addressed.

**Figure 2:**
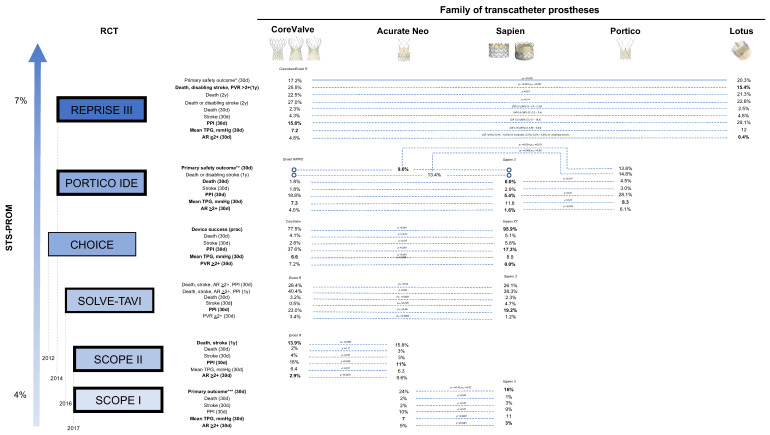
**Randomized evidence comparing different transcatheter bioprostheses.** The weight of the box outline parallels the number of patients randomized in each study. The dashed lines indicate start of enrolling for each study. Primary outcomes of available studies are reported first. Percentages in bold signal favorable outcomes with the selected prosthesis. RCT = randomized controlled trial; STS-PROM = Society of Thoracic Surgeons Predicted Risk Of Mortality. * all-cause death, stroke, life-threatening or major bleeding, stage 2/3 acute kidney injury, and major vascular complications; ** all-cause mortality, disabling stroke, life-threatening bleeding requiring transfusion, acute kidney injury requiring dialysis, or major vascular complication; *** all-cause death, any stroke, life-threatening or disabling bleeding, major vascular complications, coronary artery obstruction requiring intervention, acute kidney injury (stage 2 or 3), rehospitalization for valve-related symptoms or congestive heart failure, valve-related dysfunction requiring repeat procedure, moderate or severe prosthetic valve regurgitation, or prosthetic valve stenosis; ^ post-hoc analysis on as-treated population.

**Figure 3:**
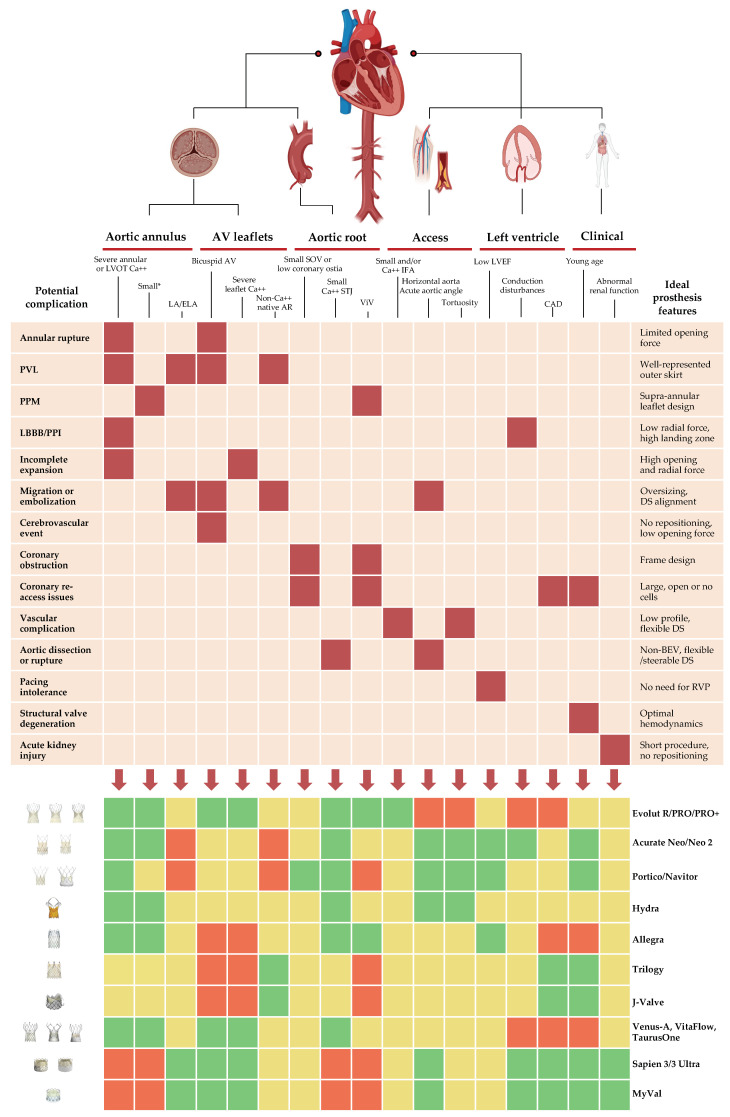
**Prosthesis tailoring for patients undergoing TAVI.** Green, yellow, and red boxes represent the strength of the support (high, moderate and low, respectively) towards utilization of that prosthesis in each setting. AV = aortic valve; BEV = balloon-expandable valve; Ca++ = calcification; CAD = coronary artery disease; DS = delivery system; ELA = extra-large annulus; IFA = ilio-femoral axis; LA = large annulus; LBBB = left bundle branch block; LVEF = left ventricular ejection fraction; LVOT = left ventricular outflow tract; PPI = permanent pacemaker implantation; PPM = prosthesis-patient mismatch; PVL = paravalvular leak; RVP = rapid ventricular pacing; SOV = sinus of Valsalva; STJ = sinotubular junction; TAVI = transcatheter aortic valve implantation; ViV = valve-in-valve. * Annulus area < 400 mm^2^ or perimeter < 72 mm.

Overall, while few observational studies have evaluated survival differences between BEV and SEV [44,45], there is currently no randomized evidence favoring one type of prosthesis in terms of mortality alone at medium-term follow-up. The main outcomes from comparative prospective randomized studies available to date are summarized in Figure 2 [24,25,27,43,46,47,48], while results from landmark randomized trials of TAVI vs. SAVR and selected head-to-head observational comparisons are included in Table 3 and Table 4 [11,12,13,14,15,49,50,51,52,53,54,55,56,57,58,59,60,61,62]. We highlight key aspects for TAVI prostheses tailoring in different clinical scenarios in Figure 3.

### 3.1. Aortic Annulus, Aortic Valve Leaflets and Aortic Root

#### 3.1.1. Annular Size

Aortic annuli dimensions at both ends of the Gaussian distribution of annular size in patients undergoing TAVI might impact periprocedural and long-term outcomes due to several reasons.

The presence of a small aortic annulus (which is defined variably in the literature, but generally includes an area <400 mm^2^ or perimeter <72 mm) is a challenge in the management of patients with severe AS, and may be associated with suboptimal forward-flow hemodynamics and worse outcomes after TAVI [63]. Data demonstrating the negative impact of prosthesis-patient mismatch (PPM) on outcomes after TAVI has been accumulating, but this remains a factor that may be underappreciated in routine clinical practice [21,64]. From our perspective, patients who have a small annulus may benefit more from implantation of a supra-annular valve, which have been shown to result in lower transprosthetic gradients, larger EOA and less PPM than intra-annular valves [21,65]. No clear intra-class difference seems to be present among different supra-annular valves, but additional factors still need to be taken into account on a case-to-case basis.

Patients with large (area ≥ 575 mm^2^ or perimeter ≥ 85 mm) and extra-large (≥683 mm^2^ or ≥94.2 mm) annuli are at a higher risk of increased risk of PVL, valve migration and valve embolization. While the latter two complications often have dramatic immediate peri-procedural effects on outcome, the impact of PVL may become more evident in medium- or long-term follow-up, even when only mild [66]. Although TAVI with 29 mm Sapien 3 and 34 mm Evolut R prostheses were shown to be safe and feasible in this group of patients [67,68], there was less overall device implant success, higher residual significant PVL, greater need for a second valve implantation and higher risk of valve embolization with SEV [68]. Of note, the MyVal device includes 30.5 and 32 mm sizes, which offers additional options in Europe for patients with extra-large annuli. The Navitor Titan valve might represent a valid alternative among SEV, even though clinical experience is scarse as of today. Overall, BEV are generally preferred in patients with large annular dimensions, even after accounting for other anatomic factors, such as valve morphology, calcification pattern, and sino-tubular junction size.

#### 3.1.2. Bicuspid Aortic Valve

Compared with tricuspid AS, bicuspid AS patients often have larger annular dimensions, more extensive calcification burden, an asymmetric orifice and co-existing dilatation of the aortic root and ascending aorta. They account for about 10% of overall TAVI candidates today. Although prospective data in this subpopulation is scarse, percutaneous treatment appears to be safe and feasible, with outcomes often comparable to tricuspid AS patients [69,70]. On the other hand, increased risk of stroke, significant PVL, device migration/embolization and annular rupture has been reported [71,72,73,74]. Newer generation BEV and MEV seem to perform better than older generation prostheses and SEV [75]. A likely explanation is the greater radial force during valve expansion, which may allow more uniform expansion in asymmetric anatomy, resulting in higher device success rate and lower significant PVL rate [76]. The Lotus and Lotus Edge had characteristics that were particularly desirable in bicuspid anatomy, including slow and controlled deployment, full repositionability, infrequent need for postdilation, and low rates of PVL. BEV are often used in this setting, but repositionability is not possible. In patients with a high burden of annular calcification or calcification distribution leading to a high risk of rupture, SEV implantation can also be considered with the understanding that the risk of significant PVL may be higher. A higher proportion of patients treated with TAVI in China have underlying bicuspid anatomy, so SEV specifically with high radial strength have been developed, including the VenusA, VitaFlow and TaurusOne valves, with promising initial results [33,34,35,36]. Until more prospective evidence is available, clear guidance of which prosthesis to choose in this setting is lacking.

#### 3.1.3. Aortic Root Calcifications

Extent and distribution of aortic root calcification influence the risk of procedural complications, including annular rupture, PVL and need for postdilation [77,78]. There are currently no randomized comparisons of different prostheses based on severity of calcification. Although the risk of incomplete prosthesis expansion in severe leaflet calcification might be reduced by predilation and/or postdilation when implanting SEV [79,80], BEV are often considered first choice to overcome the associated high resistance [81]. On the other hand, the lower opening force of an SEV may be desirable in severe annular, left ventricular outflow tract or sinotubular junction calcification since the risk of annular rupture or aortic dissection may be greater than the risk of incomplete valve expansion. This is particularly important to consider when the annulus is small or in the presence of discrete nodular calcification. Device features designed to minimize PVL, such as the pericaridal wrap of the Evolut PRO+ or the active sealing cuff of the Navitor may also be beneficial in this situation.

#### 3.1.4. Aortic Root Anatomy

Careful evaluation of aortic root anatomy is essential to minimize the risk of symptomatic coronary artery obstruction due to either coronary ostium obstruction or coronary sinus sequestration. Although rare, it is often life threatening in the absence of protection from functioning coronary artery bypass grafts. This complication occurs more often in women, and in patients with low-lying coronary ostia, shallow and shorter sinuses of Valsalva or previous surgical aortic prosthesis [82]. There is also a higher incidence in BEV compared to SEV, possibly due to the ability to reposition or retrieve most SEV designs before final deployment. Delayed coronary obstruction can also occur and has been described more commonly after SEV implantation [83]. Specific design features that may help reduce the risk of coronary obstruction include the Acurate Neo/Neo 2 stent frame’s upper crown or the JenaValve frame elements designed to hook onto the native leaflets, both of which may restrict movement of native leaflets towards the coronary ostia.

#### 3.1.5. Valve-in-Valve

There is an anticipated increase in the prevalence of valve deterioration requiring reintervention in the future due to aging of the population previously treated with SAVR and the rising numbers of TAVI procedures. ViV TAVI-in-SAVR was FDA approved using the Evolut in 2015 and Sapien in 2017. Although ViV TAVI was shown in registry data to be as safe and effective as repeat SAVR [84], it should be recognized that there are important risks of PPM and coronary obstruction. Careful procedural planning can help avoid them.

Identification of the mechanism of failure and historical hemodynamic performance of the failed prosthesis is key, since previously existing PPM may favor implantation of a supra-annular device for a ViV procedure. Additionally, higher transvalvular gradients are often seen in ViV for SAVR degeneration compared to ViV for TAVI degeneration, which may also suggest it is preferable to use a supra-annular prosthesis [85]. Certain SAVR devices (Medtronic Mosaic, Edwards Lifesciences Perimount and Magna, or Sorin Mitroflow) can undergo bioprosthetic valve fracture or modification to maximize the size and final EOA of a ViV device [86].

While there is no difference in incidence of coronary obstruction after ViV for SAVR degeneration using a BEV versus SEV [87], patients with previous TAVI using a supra-annular SEV are at increased risk of coronary sinus sequestration with ViV compared to those who had a TAVI with a BEV [88]. This is an important consideration in the lifetime management of patients and anticipated future possibility of a ViV or valve-in-valve-in-valve procedure when it comes to device selection for the initial TAVI. The introduction of Bioprosthetic Aortic Scallop Intentional Laceration to prevent Iatrogenic Coronary Artery obstruction (BASILICA) may also help allieviate some of the risk for coronary obstruction in ViV procedures [89].

Specifically in the setting of ViV for TAVI degeneration, a supra-annular SEV implanted within a BEV may offer better forward flow hemodynamics, whereas a BEV may offer greater stability and improve PVL around the original SEV [90]. Thus, it is key to understand the mechanism of index TAVI prosthesis failure in order to tackle it with a device holding the appropriate characteristics for that particular clinical scenario. It should be recognized that these statements remain speculative, and the Sapien 3 is the only currently FDA approved prosthesis for ViV after index TAVI at the time of writing this paper.

#### 3.1.6. Pure Native AR

Treatement of patients with pure severe native valve AR not amenable to conventional surgery using off-label implantation of prostheses approved for AS has been described. Whilst SAVR still represents the most appropriate management option when feasible, the choice of prosthesis can help avoid the most common complications of TAVI in this context, that is significant PVL and valve migration or embolization. In absence of significant aortic valve fibrosis or calcification for anchoring, newer generation prostheses performed better than old generation valves [91,92]. Direct comparison between BEV and SEV in this context are not available at this time. Conceptually, BEV designs with prominent outer skirts and the ability to oversize significantly may be an attractive option, while the repositionability and aortic stabilization of SEV may also make them reasonable alternatives. The recent introduction of MyVal in the European market with sizes up to 32 mm may permit treatment of a wider range of patients, especially since the annulus is often large in pure AR. The role of Navitor Titan SEV in AR is also to be defined. Dedicated prostheses with native leaflet anchoring design features have been developed to address this unmet clinical need, and include the Trilogy and J-Valve, available in Europe and China, respectively.

### 3.2. Access and Delivery

#### 3.2.1. Peripheral Vessel Calcification, Size and Tortuosity

Transvascular, especially transfemoral, is the most commonly used approach for TAVI, making size, atherosclerotic disease burden and tortuosity of peripheral vessels very important. Another factor to take into account is the diameter of the sheath (or delivery system in sheathless insertion) relative to native vessels, recognizing that most manufacturers report internal diameters as opposed to the larger maximum outer diameter (Table 1). The ratio of the sheath outer diameter to the minimal femoral artery diameter predicts vascular complications [93]. With this in mind, the very low profile of Evolut R may make it lower risk when used in patients with very small vessels (<5 mm in diameter). In patients undergoing implantation of the Sapien 3 BEV, predilation to expand the eSheath before transit with the prosthesis in severely calcified and stenotic iliofemoral arteries appears to be safe [94]. Presence of severe vessel tortuosity may favor Portico/Navitor, whose delivery system offers a greater degree of flexibility compared to other systems and can be inserted sheathless for smaller vessels [95]. Finally, intravascular lithotripsy-enabled vascular access has recently made transfemoral TAVI feasible in those who would have previously been ineligible, and should be acknowledged as part of the toolbox available to implanters [96].

#### 3.2.2. Aortic Root Angulation

A horizontal aorta (aortic angulation, defined as the angle between the virtual basal ring and the horizontal plane, ≥48–49° according to different studies) is frequently encountered and makes it more challenging to achieve adequate positioning and sealing of the prosthesis. This adverse effect on acute procedural success is seen with SEV, but not BEV, and may be particularly true for SEV with tall stent frames. A feature of the Sapien 3/3 Ultra BEV family that may partially explain this difference is the dual articulation of its steerable delivery system [97]. Among SEV, intra-class differences in device success in horizontal aortas seem to be present. The Evolut family were found to have lower device success than Acurate Neo in patients with a horizontal aorta, while the opposite was true in those without [98]. This difference may be due to the flexibility of the delivery system, which permits its leaning on the outer aortic curvature, and to the stabilization arches of the Acurate Neo that both help with alignment [99]. Enhancements with successive prosthesis iterations, such as the pericardial skirt of the Acurate Neo 2 and the more flexible capsule of the Evolut PRO+, may increase likelihood of procedural success. At this time, the Sapien 3/3 Ultra BEV should be considered first for a horizontal aorta, while Portico or Acurate Neo/Neo 2 are reasonable if contemplating a SEV or supra-annular device.

### 3.3. Left Ventricle

#### 3.3.1. Pre-Existing Conduction Disturbances

Pre-existing conduction disease increases the risk of atrioventricular block requiring permanent pacemaker implantation (PPI) in patients undergoing TAVI, which then affects both acute and long-term outcomes [22,100]. The type of prosthesis implanted affects the incidence of such complications, making device selection important [22]. The radial force of the prostheses is variable, even among those with the same mechanism of expansion, and affects impingement of the conduction system and associated conduction disturbances [22,101]. Landmark randomized trials demonstrated the overall superiority of BEV vs. SEV for PPI rate, specifically the Sapien XT vs. CoreValve [46], Sapien 3 vs. Evolut R and PRO [27,47], and Sapien 3 vs. Portico [27]. Among SEV, the Acurate Neo performed better than Evolut R and similarly to Sapien 3 in SCOPE II and I, respectively [24,25]. The cusp-overlap technique was shown to decrease PPI in TAVI with Evolut valve, but direct valve comparisons using this technique are still lacking [102]. Patients undergoing Lotus implantation had almost double the incidence of PPI than CoreValve/Evolut R in REPRISE III [43]. Overall, SEV with low radial force, such as the Acurate Neo/Neo 2, and BEV, such as the Sapien 3/3 Ultra, may be the best options for those with pre-existing conduction disturbances at high risk of PPI.

#### 3.3.2. Left Ventricular Ejection Fraction

Patients with low-flow low-gradient AS (LFLGAS) due to low left ventricular ejection fraction undergoing TAVI have worse peri-procedural, short- and long-term outcomes compared with those with normal flow AS [103,104]. These individuals may be particularly vulnerable to peri-procedural complications such as conduction abnormalities, significant PVL, and high transprosthetic gradients. The rapid ventricular pacing (RVP) commonly performed during TAVI and has also been shown to be associated with adverse outcomes, including short- and long-term mortality, when done in multiple episodes or if prolonged in this patient population [105]. Since RVP is mandatory for BEV implantation but not for SEV, this may be an important consideration for LFLGAS. On the other hand, BEV implantation may be associated with overall shorter procedure times, which may also be beneficial. Since hemodynamic stability during the entire deployment of Acurate Neo/Neo 2 SEV is expected, this may make it the best option for this patient population. Similarly, Allegra’s Permaflow technology allows flow mainteinance and hemodynamic stability during deployment.

#### 3.3.3. Coronary Artery Disease

Ease of coronary access after TAVI is becoming more and more important now that younger patients are being treated percutaneously. Although many centers prefer complete revascularization before TAVI when concomitant coronary artery disease is present, there remains the risk of further interventions needed in the future. The type of prosthesis implanted is among the most important factors to consider for ease of future coronary access. In particular, frame height, frame mesh density, leaflet commissure height and commissural tab orientation are relevant features. Relationship between prosthesis and sinus of Valsalva height and implantation depth are also important factors. One in thirteen patients experience unsuccessful coronary recannulation after TAVI, and most of them had a supra-annular SEV implanted [23,106]. Similarly, a numerical difference in successful performance of unplanned percutaneous coronary intervention after TAVI with BEV vs. SEV was recently described [107]. Commisural alignment does improve the rate of selective coronary access through supra-annular prostheses, but the risk of unsuccessful or nonselective access is still higher than with the Sapien 3 [108]. Of note, difficulty in coronary access represents an even more tangible issue after ViV procedures within supra-annular valves [109]. Overall, the Sapien 3/3 Ultra is currently the ideal prosthesis for optimal future coronary access. SEV with large cells, wide stabilization arches/tentacles, or intra-annular leaflets (such as the Portico/Navitor, Acurate Neo/Neo 2 and Hydra) should also preserve access and are reasonable alternatives.

### 3.4. Clinical Factors

#### 3.4.1. Age

Patient age is an essential parameter to consider in lifetime management of severe AS, especially since TAVI was also approved for low risk younger patients (as young as 65 years old) [17,18]. While data on long-term valve durability after surgery is available [17], long-term follow-up of patients after TAVI is scarse but promising [110]. There is currently no comparative data for long-term durability between SAVR and TAVI so far. Potentially relevant differences between valve type include a signal for increased risk of subclinical leaflet thrombosis and possibly less optimal forward flow hemodynamics after intra-annular valve implantation [21,111,112], but on the other hand, a lower risk of residual significant PVL and endocarditis after BEV implantation [45,113]. Device selection according to long-term durability remains inappropriate at this time. Other important aspects to take into account for lifelong management of younger patients include conduction disturbances, long-term impact of PPI, and restriction of coronary access after the initial TAVI as well as after a probable eventual ViV. The lower risk of PPI after BEV vs. SEV implantation may favor choice of BEV in younger patients [114], although the even lower risk of PPI after SAVR needs to be acknowledged when evaluating younger patients for aortic valve replacement [17]. Advanced planning for ViV-associated issues is particularly important even at the time of initial TAVI. We know that the leaflets and skirt of the first valve that will function as a “covered stent” after ViV is dependent on the position of the leaflets themselves [115]. Therefore, an intra-annular prosthesis might be preferred in younger patients, especially in the presence of relatively short or small sinuses. In older patients, where the risk of vascular complications and PPI may be greater, anatomy and risk specific device selection is most appropriate [116].

#### 3.4.2. Sex

Although men are at increased risk of developing AS, most AS patients over 80 years old or with small aortic annuli are women. No sex differences in device success are observed among patients undergoing TAVI, but women do suffer from higher incidence of major vascular complications and major bleeding events largely due to smaller and more tortuous iliofemoral vessels [117]. A low-profile, more flexible SEV delivery system, such as the one used for the Portico/Navitor may be preferable, especially if the aortic valve is not severely calcified [117]. When women with particularly small aortic annular dimensions are treated, a SEV with supra-annular leaflets should be considered to maximize EOA and minimize risk of PPM [21,118]. Of note, the lower coronary ostia and sinuses of Valsalva heights as well as smaller sino-tubular junction diameters that tend to be seen in women increase the risk of coronary ostia occlusion and coronary sinus sequestration [117].

#### 3.4.3. Abnormal Baseline Renal Function

The prevalence of impaired renal function in patients undergoing TAVI is significant and dependent on the specific patient population. One out of thirteen patients from the PARTNER (Placement of Aortic Transcatheter Valves) 1 trial and one out of almost 500 patients in each arm of the PARTNER 3 trials had a baseline serum creatinine ≥2 mg/dL [6,13]. Fortunately, the data suggests renal function is more likely to remain stable or improve after TAVI than worsen [119]. Nonetheless, it is important to prevent acute kidney injury in those at risk [120]. It is therefore important to note that the volume of contrast agent utilized for TAVI with BEV is less than that with SEV, so in general BEV may be preferred in the setting of renal dysfunction [24]. Alternatively, a very low or contrast-zero approach should be considered in patients with severe renal dysfunction, and seems feasible regardless of the type of prosthesis implanted [121].

#### 3.4.4. Body Size

Understanding annular and aortic root dimensions in the context of body size is important in order to appropriately select valve type and size to ensure low transprosthetic gradients and large EOA. In situations where the largest possible EOA for the annulus size is desired, supra-annular valves may be preferred [24,43,46,118]. This is particularly relevant for younger patients, who have been treated more commonly in recent years. Indeed, given that patients may undergo two or even three TAVI procedures over the course of a lifetime [84], implantation of prostheses as large as possible should be encouraged even in patients with small body size at the time of the first procedure.

### 3.5. Ease of Use

Last, but not least, streamlining of procedural steps and ease of use of a particular prosthesis are factors often underestimated. Nonetheless, adoptability of available valve systems does impact on learning curve, overall prosthesis utilization and, importantly, procedural safety and success. With expanding indications for TAVI, this will be even more true in the upcoming years, when the number of procedures and operating centers will likely increase. Most experienced operators will agree that technical failure does increase with the complexity of procedural steps, so that the more straightforward the procedure is with a particular valve system, the more it will be implemented, especially when in presence of anatomical or clinical factors not particularly stringent. Overall, given also the expanding panorama of TAVI prostheses, operator experience with a particular device will likely remain an important factor to account for when tailoring prostheses for patients undergoing TAVI.

## 4. Conclusions

As of today, no single TAVI prosthesis is capable of optimally tackling the entire spectrum of challenging scenarios encountered in the clinical practice of treating AS. Selection of the best prostheses according to individual anatomy and patient specific clinical factors is of paramount importance. Fortunately, there is a continuously advancing armamentarium of prostheses and techniques to optimize outcomes after TAVI. This review summarizes several key factors to consider in everyday practice in order to best tailor valve choice for each patient.

## 5. Future Directions

The ideal prosthesis would have the following characteristics: low profile, optimal immediate and long-term hemodynamics, excellent durability, suitability for a wide range of anatomies and minimal or absence of impingement upon the conduction system. Until this is achieved, we envision a future that has an even larger array of diverse prostheses to choose from that have specific design features to tackle particular clinical challenges. Lastly, we believe advanced tissue engineering and polymer technologies may have play a significant role in advancement of heart valve replacement therapies in the future.

## Figures and Tables

**Table 1:** Currently available TAVI prostheses.

Expansion Mechanism	Prosthesis	Manufacturer	Available	FDA Approval	CE Mark	Chinese Approval	Material		Opening Force	Radial Force	Valve Diameter (mm)	Valve Annulus Size Range
							Frame	Valve				
SEV	CoreValve	Medtronic, Minneapolis, MN, USA	No	2014	2007	−	Nitinol	Porcine pericardial	+	+	26, 29, 31	20–29
	Evolut R	Medtronic, Minneapolis, MN, USA	Yes	2015	2014	−	Nitinol	Porcine pericardial	+	++	23, 26, 29, 34	18–30
	Evolut PRO	Medtronic, Minneapolis, MN, USA	Yes	2019	2017	2022	Nitinol	Porcine pericardial	+	++	23, 26, 29	18–26
	Evolut PRO+	Medtronic, Minneapolis, MN, USA	Yes	2019	2021	−	Nitinol	Porcine pericardial	+	++	23, 26, 29, 34	18-30
	Acurate TA	Boston Scientific, Marlborough, MA, USA	No	−	2011	−	Nitinol	Porcine pericardial	−	−	S, M, L	21–27
	Acurate Neo	Boston Scientific, Marlborough, MA, USA	Yes	−	2014	−	Nitinol	Porcine pericardial	−	−	S, M, L	21–27
	Acurate Neo 2	Boston Scientific, Marlborough, MA, USA	Yes	−	2020	−	Nitinol	Porcine pericardial	−	−	S, M, L	21–27
	Portico	Abbott, Chicago, IL, USA	Yes	2021	2012	−	Nitinol	Bovine pericardial	−	−	23, 25, 27, 29	19-27
	Navitor	Abbott, Chicago, IL, USA	Yes	−	2021	−	Nitinol	Bovine pericardial	−	−	23, 25, 27, 29, 35	19–30
	Hydra	Sahajanand Medical Technologies Limited, Mumbai, India	Yes	−	2020	−	Nitinol	Bovine pericardial	+	+	22, 26, 30	18–28
	Allegra	New Valve Technology, Hechingen, Germany	Yes	−	2017	−	Nitinol	Bovine pericardial	+	+	23, 27, 31	19–28
	JenaValve	JenaValve Technology GmbH, Munich, Germany	No	−	2011	−	Nitinol	Bovine pericardial	−	−	23, 25, 27	21–27
	Trilogy	JenaValve Technology GmbH, Munich, Germany	Yes	−	2021	−	Nitinol	Bovine pericardial	−	−	23, 25, 27	21–27
	J-Valve	JC Medical Inc., Burlingame, CA, USA	Yes	−	−	2017	Nitinol	Bovine pericardial	+	+	22, 25, 28 (TF)	NA
	Venus-A	Venus Medtech Inc., Hangzhou, China	Yes	−	−	2017	Nitinol	Porcine pericardial	+	++	23, 26, 29, 32	NA
	VitaFlow	MicroPort, Shanghai, China	Yes	−	−	2019	Nitinol	Bovine pericardial	+	+	21, 24, 27, 30	21–30
	TaurusOne	Peijia Medical, Suzhou, China	Yes	−	−	2021	Nitinol	Bovine pericardial	+	+	23, 26, 29, 31	18–29
BEV	Sapien	Edwards Lifesciences, Irvine, CA, USA	No	2011	2007	−	SS	Bovine pericardial	++	+	23, 26	18–25
	Sapien XT	Edwards Lifesciences, Irvine, CA, USA	No	2014	2010	−	CoCr	Bovine pericardial	++	+	23, 26, 29	18–27
	Sapien 3	Edwards Lifesciences, Irvine, CA, USA	Yes	2015	2014	2020	CoCr	Bovine pericardial	++	+	20, 23, 26, 29	18.6–29.5
	Sapien 3 Ultra	Edwards Lifesciences, Irvine, CA, USA	Yes	2018	2018	−	CoCr	Bovine pericardial	++	+	20, 23, 26	18.6–26.4
	MyVal	Meril, Vapi, Gujarat, India	Yes	−	2019	−	NiCo	Bovine pericardial	++	−	20–32 (every 1.5 mm)	18–32
MEV	Lotus	Boston Scientific, Marlborough, MA, USA	No	−	2013	−	Nitinol	Bovine pericardial	++	+++	23, 25, 27	20–27
	Lotus Edge	Boston Scientific, Marlborough, MA, USA	No	2019	2016	−	Nitinol	Bovine pericardial	++	+++	21, 23, 25, 27, 29	18–29

BEV = balloon-expandable valve; CE = Conformitée Européenne; CoCr = cobalt-chromium; FDA = Food and Drug Administration; MEV = mechanically-expandable valve; NiCo = nickel-cobalt; SEV = self-expandable valve; SS = stainless steel. Commissural alignment feasibility is described as ++ (easy), + (possible), − (stochas-tic).

**Table 2:** Currently available TAVI prostheses-Additional information.

Expansion Mechanism	Prosthesis	Valve Height (mm)	Leaflet Position	Frame Cell Size	Outer Seal	Access	Sheath			Delivery System			Repositionable	Retrievable	Commissural Alignment
							ID/OD (French)	Integrated	Expandable	OD (French)	Flexible	Steerable			
SEV	CoreValve	53–55	Supra-annular	+	−	TV, TAo	Variable	−	Variable	18	−	−	+	+	+
	Evolut R	45–46	Supra-annular	+	−	TV/TAo	14/18, 16/20 (34 mm)	+	−	14, 16	−	−	+	+	+
	Evolut PRO	45	Supra-annular	+	+	TV/TAo	16/20	+	−	16	−	−	+	+	+
	Evolut PRO+	45–46	Supra-annular	+	++	TV	14/18, 16/20 (34 mm)	+	−	14, 16	−	−	+	+	+
	Acurate TA	44–46	Intra-annular	+++	−	TA	−	−	−	28	−	−	−	−	+
	Acurate Neo	48–51	Supra-annular	+++	+	TV, TA	14/23	−	+	18	+	−	−	−	+
	Acurate Neo 2	48–51	Supra-annular	+++	++	TV, TA	14/23	−	+	14	+	−	−	−	+
	Portico	47–51	Intra-annular	++	+	TV, TAo	14/18, 15/19 (27, 29 mm)	+	−	18, 19	++	−	+	+	+
	Navitor	47–48	Intra-annular	++	++	TV	14/18, 15/19 (27, 29, 35 mm)	+	−	14, 15	++	−	+	+	+
	Hydra	51–55	Supra-annular	+++	+	TV	18/NA	−	−	18	+	−	+	+	+
	Allegra	37–43	Supra-annular	+	−	TV	18/20.4	−	−	18	+	−	+	+	+
	JenaValve	NA	Supra-annular	+++	−	TA	−	−	−	32	−	−	+	−	++
	Trilogy	NA	Supra-annular	++	+	TV	18	−	−	18	+	+	+	−	++
	J-Valve	NA	Intra-annular	NA	−	TV/TA	NA	−	−	18	−	+	−	−	++
	Venus-A	NA	Supra-annular	+	+	TV	NA	NA	NA	19	NA	NA	+	−	NA
	VitaFlow	NA	Supra-annular	++	++	TV	NA	NA	NA	16/18	NA	NA	+	−	NA
	TaurusOne	NA	Supra-annular	++	+	TV	NA	NA	NA	18	NA	NA	+	−	NA
BEV	Sapien	14–16	Intra-annular	+	−	TV/TA/TAo	22/26, 24/28 (26 mm)	−	+	22, 24	−	−	−	−	−
	Sapien XT	14–19	Intra-annular	+	−	TV/TA/TAo	16/20, 18/22 (26 mm), 20/24 (29 mm)	−	+	16, 18, 20	−	−	−	−	−
	Sapien 3	15–22	Intra-annular	++	+	TV/TA/TAo	14/17.4, 16/20 (29 mm)	−	+	18, 21	−	+	−	−	−
	Sapien 3 Ultra	15–20	Intra-annular	++	++	TV	14/17.4	−	+	18	−	+	−	−	−
	MyVal	17–21	Intra-annular	++	++	TV	14/17.4	−	+	14	−	+	−	+	−
MEV	Lotus	19	Intra-annular	+	++	TV, TAo	18/22, 20/24 (25, 27 mm)	−	−	18, 20	−	−	++	++	−
	Lotus Edge	19	Intra-annular	+	++	TV, TAo	15/23.7	−	+	22	+	−	++	++	−

BEV = balloon-expandable valve; ID = internal diameter; MEV = mechanically-expandable valve; OD = outer diameter; SEV = self-expandable valve; TA = transapical; TAo = transaortic; TV = transvascular. Commissural alignment feasibility is described as ++ (easy), + (possible), − (stochas-tic).

**Table 3:** Clinical outcomes after TAVI with devices form selected randomized controlled trials and registries.

Expansion Mechanism	Prosthesis	Source	Type	Mean STS-PROM	Publication Year	N	Mortality %				MI %					Stroke %					Major Bleeding %			MVC %		
							30d	1y	2y	5y	IH	30d	1y	2y	5y	IH	30d	1y	2y	5y	IH	30d	1y	IH	30d	1y
SEV	CoreValve	**CHOICE**	RCT	6.2	2015	120		12.8					0.9					3.4					14.5			12.0
	CoreValve	NOTION	RCT	2.9	2015; 2019	145		4.9		27.6			3.5		7.6			2.9		9.0		11.3			5.6	
	CoreValve	SURTAVI	RCT	4.4	2017	864		6.7	11.4				2.0	2.8				5.4	6.2			12.2			6.0	
	CoreValve	**REPRISE III**	RCT	6.9	2018; 2019	305		11.9	27.0				3.2	6.1				9.4	11.4			10.9			5.3	
	Evolut R	Evolut Low Risk	RCT	1.9	2019; 2022	734		2.4	4.5				1.7	2.2				4.1	4.9			3.2	4.5		3.8	3.8
	Evolut R	**SCOPE II**	RCT	2.7	2020	398		9					1					6				3 *		NA		
	Evolut R	**PORTICO-IDE**	RCT	6.2	2020	110		15.4	26.1		NA							5.5	9.1			4.5			7.2	
	Evolut PRO	**NEOPRO**	Registry	5.3	2019	288				2.2		0.4					2.5					3.9			3.5	
	Acurate Neo	**Husser et al.**	Registry	18 **	2017	311	2.3					NA					2.3					4.2			10.3	
	Acurate Neo	SAVI TF	Registry	6.0	2018	1000		8.0					1.3					3.5					2.0	NA		
	Acurate Neo	**SCOPE I**	RCT	3.7 ***	2019	372	2					1					2					11			8	
	Acurate Neo	**NEOPRO**	Registry	5.0	2019	1263	3.0					0.6					2.0					6.8			6.0	
	Acurate Neo	**SCOPE II**	RCT	3.0	2020	398		13					1					5				2 *		NA		
	Portico	PORTICO-I	Registry	5.8	2018; 2018	941	2.7	12.1				1.6	2.5				2.6	4.1				8.5	8.7		5.5	5.7
	Portico	**PORTICO-IDE**	RCT	6.3	2020	375		14.6	22.7		NA							4.5	6.3			5.4			9.6	
BEV	Sapien XT	**CHOICE**	RCT	5.6	2015	121		17.4					0.8					9.1					21.5			11.6
	Sapien XT	PARTNER 2A	RCT	5.8	2016; 2020	1011		14.5		47.9			2.5		11.1			8.0		15.3		10.4			7.9	
	Sapien 3	Sapien 3 Intermediate Risk	Registry	5.2	2016	1077		4.0					0.3					4.6					4.6			6.1
	Sapien 3	**Husser et al.**	Registry	18 **	2017	622	2.3				NA						2.3					4.2			10.3	
	Sapien 3	Low risk TAVR	Registry	1.8	2018; 2021	200	0	3.0	4.2		0	0	1.0	1.1		0	0.5	2.1	4.3		2.5	3.0		2.5	3.0	
	Sapien 3	PARTNER 3	RCT	1.9	2019; 2021	496		1.0	2.5				1.2					1.2	3.5			3.6			2.2	
	Sapien 3	**SCOPE I**	RCT	3.4 ***	2019	367				1					0		3					9			5	
	Sapien 3	**PORTICO-IDE**	RCT	6.5	2020	206		8.4		15.6		NA						6.0	8.0			4.4			7.3	
MEV	Lotus	**REPRISE III**	RCT	6.7	2018; 2019	607		11.9	22.8				3.2	6.1				7.0	8.4			12.8			7.0	
	Lotus	RESPOND/Extension	Registry	6.0	2017; 2019	996	2.6	11.7				0.3	1.9				3.0	4.9				2.6 *	3.6		3.4	3.4

Sources in bold represent comparative studies between different transcatheter prostheses. Reported type of prosthesis is either the only one implanted or the most represented prosthesis among the different generations implanted. * Life-threatening or major bleeding (BARC 3b or more); ** Logistic EuroSCORE I; *** median. AKI = acute kidney injury; BEV = balloon-expandable valve; IH = in-hospital; MEV = mechanically-expandable valve; MI = myocardial infarction; PPI = permanent pacemaker implantation; RCT = randomized controlled trial; SEV = self-expandable valve; STS-PROM = Society of Thoracic Surgery-Predicted Risk Of Mortality.

**Table 4:** Clinical outcomes after TAVI with devices form selected randomized controlled trials and registries–Additional endpoints.

Expansion	Prosthesis	Source	Type	AKI %			PPI %			Gradient					EOA cm^2^				Severe PPM	Mild PVL					Moderate or More PVL				
				IH	30d	1y	IH	30d	1y	IH	30d	1y	2y	5y	30d	1y	2y	5y	30d	IH	30d	1y	2y	5y	IH	30d	1y	2y	5y
SEV	CoreValve	**CHOICE**	RCT	NA					38.0			38.0			1.8 (0.6)				NA			39.6					12.1		
	CoreValve	NOTION	RCT		0.7			34.1			34.1					1.7		1.7	NA			55.4		52.9			15.7		8.2
	CoreValve	SURTAVI	RCT		1.7			25.9			25.9					2.2 (0.6)	2.2 (0.7)		NA			31.9	32.8				5.3	3.9	
	CoreValve	**REPRISE III**	RCT		3.6			19.6			19.6					2.0 (0.5)	1.8 (0.5)		NA			38.8					6.8	3.8	
	Evolut R	Evolut Low Risk	RCT		0.9			17.4			17.4					2.3 (0.7)	2.2 (0.5)		1.1			33.1	26.6				3.7	1.7	
	Evolut R	**SCOPE II**	RCT	NA	9			18			18				1.8 (0.5)	1.8 (0.6)			NA		52.2			35.9		2.9	3.3		
	Evolut R	**PORTICO-IDE**	RCT		2.7			18.8							18.8				1.9 (0.4)		1.8 (0.5)		4	NA		4.0		0	
	Evolut PRO	**NEOPRO**	Registry		2.1			13.2			13.2			2.5					NA	42.1					5.8				
	Acurate Neo	**Husser et al.**	Registry		3.2			10.2						10.2					4.2			NA		4.8				NA	
	Acurate Neo	SAVI TF	Registry	NA					9.9			9.9				1.8 (0.4)			NA					40.4			1.8		
	Acurate Neo	**SCOPE I**	RCT		8			10			10			2	1.7				NA		50.1					9.4			
	Acurate Neo	**NEOPRO**	Registry		3.1			8.8			8.8			2.0					NA	56.9					5.2				
	Acurate Neo	**SCOPE II**	RCT	NA				11			11				1.7 (0.5)	1.8 (0.5)			6		63.2			57.3		9.6	4.0		
	Portico	PORTICO-I	Registry		3.0	4.2		18.7	21.3		18.7	21.3		2.6	1.8 (0.5)	1.7 (0.5)			NA		67.6	67.9				3.9	2.6		
	Portico	**PORTICO-IDE**	RCT		3.0			28.1							28.1				1.8 (0.5)		1.9 (0.5)		NA	NA		6.1		5.2	
BEV	Sapien XT	**CHOICE**	RCT	NA	17.4				23.4			23.4			1.7 (0.4)				NA			40.4					1.1		
	Sapien XT	PARTNER 2A	RCT		1.3			8.5			8.5					1.6 (0.5)		1.5	NA			23.2		17.0			3.4		4.1
	Sapien 3	Sapien 3 Intermediate Risk	Registry			0.5			10.2			10.2				1.7 (0.4)			NA			30.3					0		
	Sapien 3	**Husser et al.**	Registry		3.2			10.2							10.2							10.3	NA		NA				
	Sapien 3	Low risk TAVR	Registry	0	0		5.0	6.5	7.3	5.0	6.5	7.3	0	0.5	1.6 (0.4)	1.7 (0.5)	1.7 (0.5)		NA		31.1					0.5	1.5	0	
	Sapien 3	PARTNER 3	RCT		0.4			7.9	9.1		7.9	9.1				1.7 (0.02)	1.7 (0.37)		8.3		25.1	24.3	20.0			0.8	0.8	0.5	
	Sapien 3	**SCOPE I**	RCT		7			9			9			3	1.5				NA		31.1					2.8			
	Sapien 3	**PORTICO-IDE**	RCT		0.5			5.4						5.4		8.0		1.6 (0.4)		1.6 (0.5)		NA	NA			1.6		0.8	
MEV	Lotus	**REPRISE III**	RCT		2.5			35.5			35.5					1.6 (0.5)	1.5 (0.5)		NA			11.3					0.9	0.3	
	Lotus	RESPOND/Extension	Registry		1.7	1.7		34.6	37.2		34.6	37.2		3.0	1.8 (0.4)	1.8 (0.4)			NA		7.7	5.5				0.3	0.4		

Reported type of prosthesis is either the only one implanted or the most represented prosthesis among the different generations implanted. Sources in bold represent comparative studies between different transcatheter prostheses. AKI = acute kidney injury; BEV = balloon-expandable valve; IH = in-hospital; MEV = mechanically-expandable valve; MI = myocardial infarction; PPI = permanent pacemaker implantation; RCT = randomized controlled trial; SEV = self-expandable valve; STS-PROM = Society of Thoracic Surgery-Predicted Risk Of Mortality.

## Data Availability

Not applicable.

## Abbreviations

AS	aortic stenosis
AR	aortic regurgitation
BEV	balloon-expandable valve
EOA	effective orifice area
PPM	prosthesis-patient mismatch
MEV	mechanically expandable valve
PVL	paravalvular leak
RVP	rapid ventricular pacing
SAVR	surgical aortic valve replacement
SEV	self-expandable valve
TAVI	transcatheter aortic valve implantation
ViV	valve-in-valve
